# Quantitative analysis of performance on a progressive-ratio schedule: effects of reinforcer type, food deprivation and acute treatment with Δ^9^-tetrahydrocannabinol (THC)

**DOI:** 10.1016/j.beproc.2015.01.014

**Published:** 2015-04

**Authors:** C.M. Olarte-Sánchez, L. Valencia-Torres, H.J. Cassaday, C.M. Bradshaw, E. Szabadi

**Affiliations:** a Psychopharmacology Section, Division of Psychiatry, University of Nottingham, UK; b School of Psychology, University of Nottingham, UK

**Keywords:** Progressive-ratio schedule, Mathematical Principles of Reinforcement, Mathematical model, Food deprivation, Sucrose, Corn oil, Δ^9^-Tetrahydrocannabinol, Incentive value, Rat

## Abstract

Rats’ performance on a progressive-ratio schedule maintained by sucrose (0.6 M, 50 μl) and corn oil (100%, 25 μl) reinforcers was assessed using a model derived from Killeen’s (1994) theory of schedule-controlled behaviour, ‘Mathematical Principles of Reinforcement’. When the rats were maintained at 80% of their free-feeding body weights, the parameter expressing incentive value, *a*, was greater for the corn oil than for the sucrose reinforcer; the response-time parameter, *δ*, did not differ between the reinforcer types, but a parameter derived from the linear waiting principle (*T*_0_), indicated that the minimum post-reinforcement pause was longer for corn oil than for sucrose. When the rats were maintained under free-feeding conditions, *a* was reduced, indicating a reduction of incentive value, but *δ* was unaltered. Under the food-deprived condition, the CB1 cannabinoid receptor agonist Δ^9^-tetrahydrocannabinol (THC: 0.3, 1 and 3 mg kg^−1^) increased the value of *a* for sucrose but not for corn oil, suggesting a selective enhancement of the incentive value of sucrose; none of the other parameters was affected by THC. The results provide new information about the sensitivity of the model’s parameters to deprivation and reinforcer quality, and suggest that THC selectively enhances the incentive value of sucrose.

## Highlights

• Rats were trained under a progressive-ratio schedule of sucrose or corn oil reinforcement.
• A mathematical model of progressive-ratio schedule performance was used to analyse the data.
• The model’s incentive value parameter was greater for the corn oil than for the sucrose reinforcer.
• Removal of the food restriction condition resulted in reduction of the incentive value parameter.
• Δ^9^-Tetrahydrocannabinol selectively increased the incentive value of sucrose but not corn oil.

## 1 Introduction

In ratio schedules of reinforcement, the subject is required to emit a specified number of responses, *N*, to obtain a reinforcer. In progressive-ratio schedules, *N* is systematically increased, usually from one reinforcer to the next (Hodos, 1961; Stafford and Branch, 1998), but sometimes after batches of two or more reinforcers (Baunez et al., 2002; Randall et al., 2012) or between successive sessions (Griffiths et al., 1978; Czachowski and Samson, 1999). Performance on progressive-ratio schedules is characterised by rapid responding under low ratios which peters out as *N* is increased. The ratio at which the subject stops responding, the breakpoint, is widely regarded as a measure of the subject’s motivation or the incentive value of the reinforcer (Hodos, 1961; Hodos and Kalman, 1963 for review, see Ping-Teng et al., 1996; Killeen et al., 2009).

Despite its widespread use, several authors have expressed doubts about the specificity of the breakpoint (Arnold and Roberts 1997; Killeen et al., 2009; Bradshaw and Killeen, 2012), pointing out that it is sensitive not only to changes in the incentive properties of reinforcers (Rickard et al., 2009; Gosnell et al., 2010) but also to non-motivational manipulations such as changes in the response requirement (Skjoldager et al., 1993; Aberman et al., 1998) and the ratio step size (Covarrubias and Aparicio, 2008). It has also been noted that the breakpoint shows considerable variability, being derived from a single point in time while data from the rest of the session are ignored, and that its definition is arbitrary, there being no consensus as to the time that must elapse without a response before responding may be said to have stopped (Arnold and Roberts, 1997; Killeen et al., 2009).

Many of the shortcomings of the breakpoint may be avoided by the use of quantitative models of performance on progressive-ratio schedules, for example the model recently proposed by Bradshaw and Killeen (2012). This model was derived from Killeen’s (1994) general theory of schedule-controlled behaviour, the Mathematical Principles of Reinforcement (MPR), according to which schedule-controlled responding is determined by an excitatory effect of reinforcers on behaviour, biological constraints on responding, and the efficiency with which schedules couple responses to reinforcers. In addition, the progressive-ratio model invokes the linear waiting principle (Wynne et al., 1996) to predict the escalating duration of the post-reinforcement pause in successive ratios, thereby yielding a dynamic account of performance on this schedule. The linear waiting principle expresses the finding that the post-reinforcement pause on trial *i*, *T*_P*,i*_, is linearly related to the total inter-reinforcement interval on trial *i*-1, *T*_TOT,*i*-1_:

(1) $$T_{\text{P},i}=T_{0}+kT_{TOT,i-1}$$

where the parameters *T*_0_ and *k* represent the minimum post-reinforcement pause and the slope of the linear waiting function. The progressive-ratio model contains two key equations that define running response rate, *R*_RUN_, and overall response rate, *R*_OVERALL_:

(2) $$R_{\text{RUN},i}=\frac{1}{\delta (1+T_{\text{TOT},\;i-1}/a)}$$

(3) $$R_{\text{OVERALL},i}=N_{i}/T_{\text{TOT},i}$$

The parameter *a* (‘specific activation’), which is defined as the duration of behavioural activation induced by a single reinforcer, is regarded as an index of incentive value. *δ* is the minimum time needed to execute a response (the reciprocal of the maximum response rate), and is regarded as a measure of the biological limitations on responding (Killeen, 1994; Reilly, 2003; Covarrubias and Aparicio, 2008; Sanabria et al., 2008; Bradshaw and Killeen, 2012).

Several lines of evidence support these interpretations of *a* and *δ*. Consistent with the notion that *a* is an index of incentive value, it has been found that this parameter is monotonically related to the volume of a sucrose-solution reinforcer (Rickard et al., 2009: data re-analysed by Bradshaw and Killeen, 2012). Recently, Olarte-Sánchez et al. (2013) compared the values of *a* for corn oil and sucrose reinforcers; their findings were consistent with extant evidence that sucrose is less efficacious than corn oil on a volume-for-volume basis, but more efficacious on a calorie-for-calorie basis (Naleid et al., 2008). Valencia-Torres et al. (2014) found that diabetes induced by systemic treatment with streptozotocin was associated with a reduction of *a*, consistent with an antihedonic effect of this treatment (Nefs et al., 2012). D_1_ and D_2_ dopamine receptor antagonists also reduce *a*, consistent with the purported antihedonic effect of these drugs (Olarte-Sánchez et al., 2012: data re-analysed by Bradshaw and Killeen, 2012; Olarte-Sánchez et al., 2013). Some drugs with known sedative properties, including clozapine and cyproheptadine, have been found to increase the response-time parameter *δ* (Olarte-Sánchez et al., 2012: data re-analysed by Bradshaw and Killeen, 2012).

The experiment described in this paper further explored the utility of the progressive-ratio model. The aims were firstly to examine the sensitivity of the parameters of the model to the food deprivation condition and the type of reinforcer used, and secondly to examine the effect of Δ^9^-tetrahydrocannabinol (THC), a principal constituent of cannabis resin with high affinity for CB1 cannabinoid receptors (Gaoni and Mechoulam, 1964; Howlett, 2002; Ledent et al., 1999; Matsuda et al., 1990), on the parameters of the model. Since, ex hypothesi, *a* represents the incentive value of a reinforcer, it was expected that the value of this parameter would be greater under conditions of food deprivation than under free-feeding conditions. Moreover, in view of the known orexigenic effect of THC (Abel, 1975; De Luca et al., 2012; Higgs et al., 2003; Williams et al., 1998; Williams and Kirkham, 2002a,b), it was expected that this drug would induce an increase in the value of *a*. However, in apparent conflict with the latter prediction, Olarte-Sánchez et al. (2012) recently reported that THC had no effect on the value of *a* for food-pellet reinforcers. The present experiment extended these findings by examining the effect of THC on performance on progressive-ratio schedules maintained by sucrose and corn oil reinforcers. In addition, since Olarte-Sánchez et al. (2012) analysed their data using an earlier model derived from MPR, designed to account for performance on fixed-ratio schedules (Killeen, 1994), a re-analysis of their data was carried out using the new progressive-ratio model.

## 2 Methods

The experiment was carried out in accordance with UK Home Office regulations governing experiments on living animals.

### 2.1 Subjects

Twenty-four female Wistar rats (Charles River, UK) approximately 4 months old and weighing 250–300 g at the start of the experiment were used. They were housed individually under a constant cycle of 12 h light and 12 h darkness (light on 0600–1800 h), and were maintained at 80% of their initial free-feeding body weights (see below) by providing a limited amount of standard rodent diet after each experimental session. Tap water was freely available in the home cages.

### 2.2 Apparatus

The rats were trained in operant conditioning chambers (CeNeS Ltd. Cambridge, UK) of internal dimensions 25 × 25 × 22 cm. One wall of the chamber contained a central recess covered by a hinged Perspex flap, into which a peristaltic pump delivered the liquid reinforcer (see below). An aperture located 5 cm above and 2.5 cm to one side of the recess (left for half the subjects; right for the other half) allowed insertion of a motorised retractable lever (CeNeS Ltd. Cambridge, UK) into the chamber. The lever could be depressed by a force of approximately 0.2 N. The chamber was enclosed in a sound-attenuating chest with additional masking noise generated by a rotary fan. No houselight was present during the sessions. An Acorn microcomputer programmed in Arachnid BASIC (CeNeS Ltd. Cambridge, UK) located in an adjacent room controlled the schedule and recorded the behavioural data.

### 2.3 Behavioural training

Two weeks before starting the experiment the food deprivation regimen was introduced and the rats were gradually reduced to 80% of their free-feeding body weights. They were randomly allocated to two groups that underwent training with different reinforcers: 50 μl of a 0.6 M solution of sucrose in distilled water (*n* = 12), and 25 μl of undiluted corn oil (*n* = 12). The rats were first trained to press the lever for the liquid reinforcer, and were then exposed to an fixed-ratio 1 schedule for 3 days followed by fixed-ratio 5 for a further 3 days. Thereafter, they underwent daily training sessions under the progressive-ratio schedule. The progressive-ratio schedule was based on the exponential progression: 1, 2, 4, 6, 9, 12, 15, 20, 25, 32, 40, …, derived from the formula (5 × e^0.2*n*^) − 5, rounded to the nearest integer, where *n* is the position in the ratio sequence (Roberts and Richardson, 1992). Sessions took place at the same time each day during the light phase of the daily cycle (between 0800 and 1300 h) 7 days a week. At the start of each session, the lever was inserted into the chamber; the session was terminated by withdrawal of the lever 40 min later.

### 2.4 Drug treatment

Injections of THC were given on Tuesdays and Fridays, and injections of the vehicle alone on Mondays and Thursdays; no injections were given on Wednesdays, Saturdays or Sundays. Each rat was tested five times with each dose of THC, the order of treatments being counterbalanced across animals according to a Latin square design. THC (Δ^9^-tetahydrocannabinol, obtained from Tocris Bioscience, Bristol, UK) was dissolved in a mixture of ethanol and Tween (1:1) and diluted with sterile water to give the desired concentration. It was injected intraperitoneally (2.5 ml kg^−1^; 25-gauge needle) 30 min before the start of the experimental session. The doses of THC were selected on the basis of previous experience of the effect of this drug on performance on the progressive-ratio schedule (Olarte-Sánchez et al., 2012).

### 2.5 Experimental procedure

The experiment consisted of two phases. First, while the rats were maintained at 80% of their free-feeding body weights (‘food-deprived condition’), the effect of THC (0.3, 1 and 3 mg kg^−1^) was tested. Then the rats were given free access to standard laboratory chow (RM1 rodent diet: SDS Ltd., UK) in their home cages (‘free-feeding condition’) while daily training under the progressive-ratio schedule was continued.

### 2.6 Data analysis

Overall response rate (*R*_OVERALL_) was calculated for each ratio by dividing the number of responses by the total time taken to complete the ratio, including the post-reinforcement pause, measured from the end of the preceding reinforcer delivery until the emission of the last response of the ratio (Bizo and Killeen, 1997). The first ratio (a single response) and any ratios that had not been completed at the end of the session were excluded from the analysis. Running rate (*R*_RUN_) was calculated by dividing the number of responses by the ‘run-time’ (i.e. the time taken to complete the ratio, excluding the post-reinforcement pause: Bizo et al., 2001). Post-reinforcement pause duration was measured from the end of the reinforcer delivery until the emission of the first response of the following ratio.

The breakpoint was defined as the last ratio to be completed before 5 min elapsed without any responding, or, in cases where this criterion was not met within the session, the highest completed ratio (Olarte-Sánchez et al., 2012).

The progressive-ratio model comprising Eqs. (2) and (3) was fitted to the running and overall response rate data obtained from individual rats, and estimates of the four parameters, *T*_0_, *k*, *a* and *δ*, were derived using the ‘Solver’ facility of Excel (Microsoft Corporation); goodness of the combined fit of Eqs. (2) and (3) to the overall and running response rate data was expressed as *R*^2^ (Bradshaw and Killeen 2012).

The model was fitted to the data obtained from each rat in the last ten sessions in which no active treatment was administered under the food-deprived and free-feeding conditions, and estimates of the four parameters were derived. These estimates were analysed by separate two-factor analyses of variance with reinforcer type as a between-groups factor and deprivation condition as a within-subject factor, followed, in the case of a significant interaction, by post hoc comparisons of the two groups within conditions, and the two conditions within groups, using Student’s *t*-test.

The model was also fitted to the data obtained from each rat in the sessions in which injections of THC or its vehicle were administered, and estimates of the four parameters were derived. These estimates were analysed by separate one-factor analyses of variance with treatment condition as a within-subject factor, followed, in the case of a significant effect of treatment, by comparison of each dose of THC with the vehicle-alone treatment using Dunnett’s test. The effect sizes revealed by the analyses of variance were expressed as partial *η*^2^ (*η*^2^*_p_*).

The same statistical methods as were used to analyse parameters of the model were also used to analyse the breakpoints.

In addition to the results of the present experiment, the same analytical methods were used to re-analyse the data reported by Olarte-Sánchez et al. (2012) on the effect of THC on performance maintained by a progressive-ratio schedule of food-pellet reinforcement. These data were obtained from 12 female Wistar rats maintained under the same conditions, trained under the same progressive-ratio schedule, and tested with the same doses of THC as those used in the present experiment. A significance criterion of *p *< 0.05 (two-tailed) was adopted in all statistical analyses.

## 3 Results

### 3.1 Comparison of performance maintained by sucrose and corn oil reinforcers

**Fig. 1:**
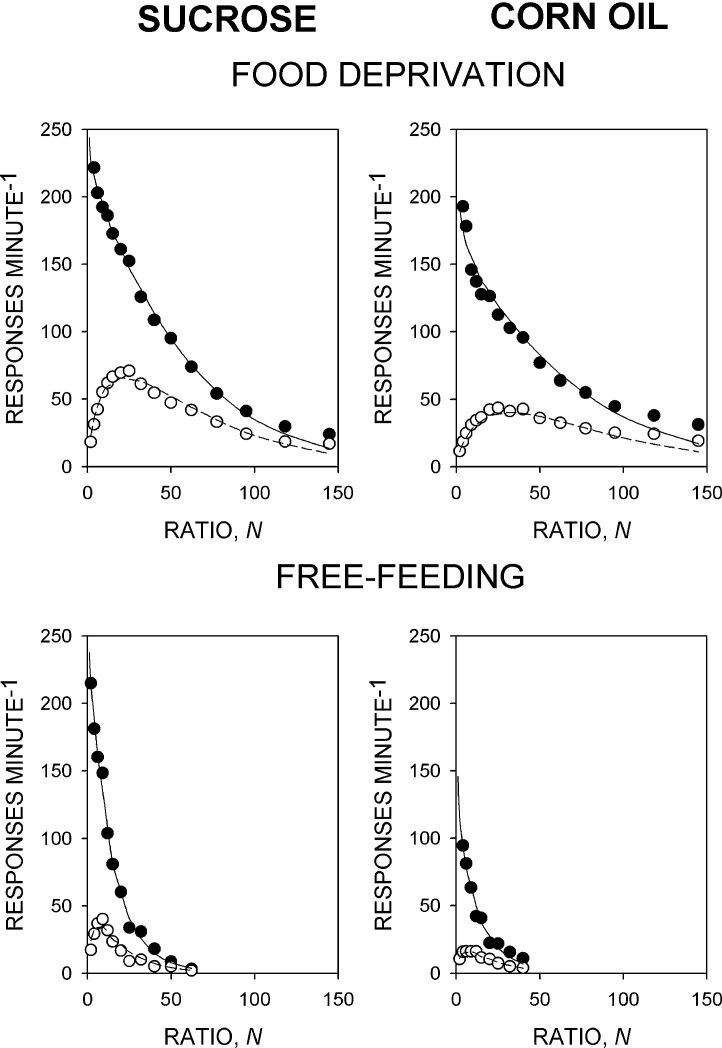
Performance on the progressive-ratio schedule maintained by the sucrose-solution and corn oil reinforcers (left and right columns) under the food-deprived and free-feeding conditions (upper and lower rows). Ordinates, response rate; abscissae, response/reinforcer ratio, *N*. Points are group mean data: filled symbols indicate running response rate, unfilled symbols overall response rate. The curves are best-fit functions defined by Eqs. (2) and (3).

Fig. 1 shows the mean response rate data from the two groups in sessions in which no drug treatment was administered under the food-deprived and free-feeding conditions. In both groups and under both conditions, running response rate declined monotonically towards zero, whereas overall response rate rose to a peak before declining towards zero. Under the food-deprived condition, the peak of the overall response rate function was lower and the slope of the declining phase shallower in the corn oil-reinforced group than in the sucrose-reinforced group. In both groups response rates declined more steeply under the free-feeding condition than under the food-deprived condition. The progressive-ratio model provided a good description of the group mean overall and running response rate data obtained from both groups (sucrose-reinforced group: *R*^2^ = 0.995 [food-deprived], 0.994 [free-feeding]; corn oil-reinforced group: *R*^2^ = 0.983 [food-deprived], 0.988 [free-feeding]).

**Fig. 2:**
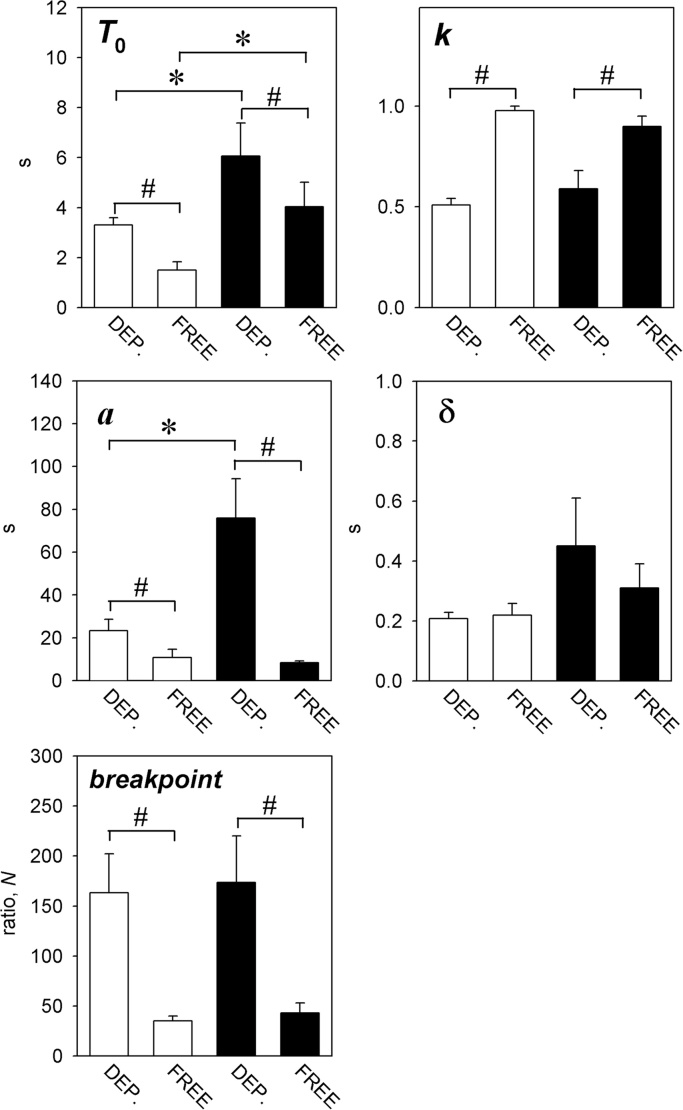
Parameters of the progressive-ratio model, and the breakpoint, for performance maintained by the sucrose-solution (empty columns) and corn oil (filled columns) reinforcers under the food-deprived (DEP) and free-feeding (FREE) conditions. Columns show group mean values + SEM. Significant differences are denoted by horizontal lines: differences between reinforcer types, *p < 0.05; differences between deprivation conditions, #*p* < 0.05. *T*_0_ was greater for corn oil than for sucrose under both deprivation conditions, and was greater under the food-deprived condition than under the free-feeding condition for both reinforcer types. *k* was greater under the free-feeding condition than under the food-deprived condition for both reinforcer types, but did not differ significantly between reinforcer types. *a* was greater under the food-deprived condition than under the free-feeding condition for both reinforcer types, and was greater for corn oil than for sucrose only under the food-deprived condition. The value of *δ* did not differ significantly between reinforcer types or deprivation conditions. The breakpoint was higher under the food-deprived condition than under the free-feeding condition for both reinforcer types.

Fig. 2 shows the mean (+SEM) estimates of the parameters of the model derived from the individual rats in the two groups under the two deprivation conditions. The data from one rat in the corn oil-reinforced group which did not respond reliably under the free-feeding condition were omitted, leaving 11 rats in the corn oil-reinforced group and 12 in the sucrose-reinforced group. Analysis of variance of the values of *T*_0_ showed significant main effects of reinforcer type [*F*(1,21) = 9.1, *p *< 0.01, *η*^2^*_p_* = 0.32] and deprivation condition [*F*(1,21) = 9.6, *p *< 0.01, *η*^2^*_p_* = 0.30], reflecting the higher values of this parameter obtained under the food-deprived than the free-feeding condition, and the lower values seen in the sucrose-reinforced group compared to the corn oil-reinforced group; the interaction was not statistically significant [*F *< 1]. In the case of *k*, there was a significant main effect of deprivation condition [*F*(1,21) = 47.9, *p *< 0.001; *η*^2^*_p_* = 0.69], reflecting higher values of this parameter seen under the free-feeding condition than under the food-deprived condition; there was no significant main effect of group [*F *< 1] and no significant interaction [*F*(1,21) = 2.1, NS, *η*^2^*_p_* = 0.16]. In the case of *a*, there were significant main effects of both reinforcer type [*F*(1,21) = 19.5, *p *< 0.001, *η*^2^*_p_* = 0.69] and deprivation condition [*F*(1,21) = 6.5, *p *< 0.05, *η*^2^*_p_* = 0.24], and a significant interaction effect [*F*(1,21) = 9.1, *p *< 0.05; *η*^2^*_p_* = 0.30]. Multiple comparisons revealed that the free-feeding condition was associated with a reduction of the value of *a*, compared to the food-deprived condition, in the case of both reinforcer types. Under the food-deprived condition, the value of *a* was greater for corn oil than for sucrose; however, under the free-feeding condition, there was no significant difference between the values of *a* for the two reinforcers. In the case of *δ*, analysis of variance revealed no significant main effect of either reinforcer type [*F*(1,21) = 4.1, NS, *η*^2^*_p_* = 0.16] or deprivation condition [*F *< 1], and no significant interaction [*F *< 1].

Also shown in Fig. 2 are the breakpoint data. Analysis of variance showed a significant main effect of deprivation condition [*F*(1,21) = 23.8, *p *< 0.001, *η*^2^*_p_* = 0.52], reflecting the higher breakpoints obtained under the food-deprived condition than under the free-feeding condition in the case of both the sucrose and the corn oil reinforcer. There was no significant main effect of reinforcer type [*F *< 1] and no significant interaction [*F *< 1].

### 3.2 Effect of THC on performance under the progressive-ratio schedule

#### 3.2.1 Sucrose-reinforced group

**Fig. 3:**
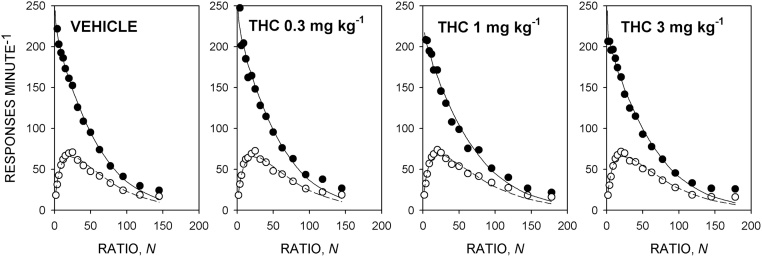
Effect of THC 0.3, 1 and 3 mg kg^−1^ on performance on the progressive-ratio schedule maintained by the sucrose-solution reinforcer under the food-deprived condition. Ordinates, response rate; abscissae, response/reinforcer ratio, *N*. Points are group mean data: filled symbols indicate running response rate, unfilled symbols overall response rate. The curves are best-fit functions defined by Eqs. (2) and (3).

Fig. 3 shows the group mean response rate data and Table 1 shows the mean ± SEM values of the parameters of the model derived from the individual rats. There was no significant effect of THC on the value of *T*_0_ [*F*(3,33) = 1.9, NS, *η*^2^*_p_* = 0.15] or *k* [*F*(3,33) = 1.4, NS, *η*^2^*_p_* = 0.11]. There was a significant effect of treatment on *a* [*F*(3,33) = 2.9, *p *< 0.05, *η*^2^*_p_* = 0.21]; the linear contrast effect was significant [*F*(1,11) = 6.7, *p *< 0.05, *η*^2^*_p_* = 0.38]. Multiple comparisons showed that the value of *a* was significantly increased by THC 1 and 3 mg kg^−1^ compared to the vehicle-alone treatment, reflecting the somewhat shallower slopes of the descending limbs of the response rate functions obtained with these doses (Fig. 3). THC had no significant effect on the value of *δ* [*F*(3,33) = 1.7, NS, *η*^2^*_p_* = 0.14]. There was no significant effect of THC on the breakpoint [*F*(3,33) = 1.4, NS, *η*^2^*_p_* = 0.12].

#### 3.2.2 Corn oil-reinforced group

**Fig. 4:**
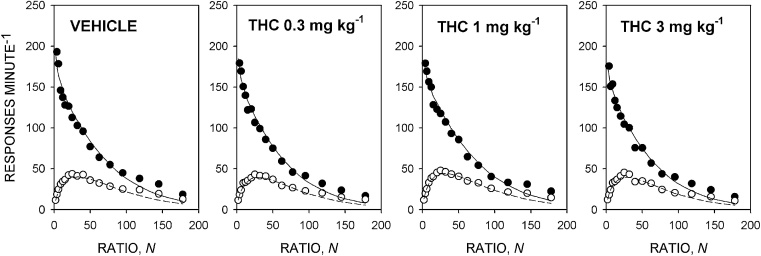
Effect of THC 0.3, 1 and 3 mg kg^−1^ on performance on the progressive-ratio schedule maintained by the corn oil reinforcer under the food-deprived condition. Ordinates, response rate; abscissae, response/reinforcer ratio, *N*. Points are group mean data: filled symbols indicate running response rate, unfilled symbols overall response rate. The curves are best-fit functions defined by Eqs. (2) and (3).

Fig. 4 shows the group mean response rate data and Table 2 shows the mean ± SEM values of the parameters of the model derived from the individual rats. THC had no significant effect on any of the parameters of the model [*T*_0_: *F *< 1; *k*: *F*(3,33) = 1.5, NS, *η*^2^*_p_* = 0.12; *a*: *F *< 1; *δ*: *F *< 1], or on the breakpoint [*F*(3,33) = 2.9, NS, *η*^2^*_p_* = 0.22].

#### 3.2.3 Food pellet-reinforced group (re-analysis of data reported by Olarte-Sánchez et al., 2012)

**Fig. 5:**
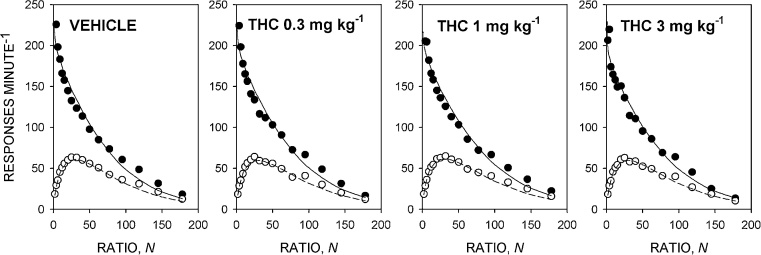
Effect of THC 0.3, 1 and 3 mg kg^−1^ on performance on the progressive-ratio schedule maintained by food-pellet reinforcer under the food-deprivation condition (re-analysis of data reported by Olarte-Sánchez et al., 2012), Ordinates, response rate; abscissae, response/reinforcer ratio, *N*. Points are group mean data: filled symbols indicate running response rate, unfilled symbols overall response rate. The curves are best-fit functions defined by Eqs. (2) and (3).

Fig. 5 shows the group mean response rate data and Table 3 shows the mean ± SEM values of the parameters of the model derived from the individual rats. THC had no significant effect on any of the parameters of the model [*T*_0_: *F*(3,33) = 1.5, NS, *η*^2^*_p_* = 0.12; *k*: *F*(3,33) = 1.8, NS, *η*^2^*_p_* = 0.14; *a*: *F *< 1; *δ*: *F*(3,33) = 1.3, NS, *η*^2^*_p_* = 0.10], or on the breakpoint [*F *< 1].

## 4 Discussion

In agreement with previous findings (Bezzina et al., 2015; Olarte-Sánchez et al., 2013; Valencia-Torres et al., 2014 also earlier data re-analysed by Bradshaw and Killeen, 2012), the present results indicate that operant behaviour maintained by progressive-ratio schedules is well described by the mathematical model of performance on this schedule (see Section 1). The results also provide new information about the sensitivity of the four parameters of the model to schedule manipulations and acute treatment with THC, a putative orexigenic drug.

### 4.1 Effect of deprivation level

The values of *a* were substantially reduced when the rats were tested under the free-feeding condition, compared to the values obtained under the food-deprived condition, indicating a reduction of the incentive values of both reinforcers when home cage feeding was not restricted.

It has, of course, been known for many years that food deprivation enhances the efficacy of food reinforcers (Clark, 1958; Hillman et al., 1953; Horenstein, 1951; Skinner, 1936). However, although most current theories of schedule-controlled behaviour (e.g. Herrnstein, 1970; Killeen, 1994) assume that deprivation enhances reinforcer value, the exact form of this relationship remains unknown. Herrnstein’s (1970) response-strength equation defines a hyperbolic relation between response rate, *R*, and reinforcement rate, *r*, thus:

(4) $$R=\frac{R_{\max}\times r}{K_{\text{H}}+r}$$

where *R*_max_ and *K*_H_ are free parameters.^1^ Herrnstein (1970, 1974) interpreted *K*_H_ (*r*_0_ in his notation) as the rate of extraneous (unobserved) reinforcement, expressed in units of the reference (food) reinforcer. According to this interpretation, the finding that an increase in the severity of food deprivation causes a reduction of the value of *K*_H_ for food-reinforced responding (Bradshaw et al., 1983; Heyman and Monaghan, 1987) implies that deprivation enhances the efficacy or value of food reinforcers. Killeen’s (1994) MPR theory also assumes that deprivation level is a determinant of reinforcer value, where value (*a*) is defined as the duration of behavioural activation induced by a single reinforcer. The present finding of a reduction of *a* following a reduction of the severity of food deprivation is clearly in accord with this assumption. However, it is important to emphasise that neither Herrnstein’s (1970) nor Killeen’s (1994) theory makes specific predictions about the form of the relation between the level of deprivation and reinforcer value. Further analysis based on systematic manipulation of deprivation conditions is needed to address this issue.

The value of *δ* did not differ between the food-deprived and free-feeding conditions, suggesting that the deprivation condition did not affect the motor aspects of performance. However the two parameters expressing the linear waiting principle, *T*_0_ and *k*, did differ between the two conditions, the value of *T*_0_ being smaller and that of *k* larger under the food-deprived condition than under the free-feeding condition. There do not appear to have been any systematic investigations of the sensitivity of the linear waiting function to the level of food deprivation; this may be an issue worth pursuing in future experiments, using procedures such as the response-initiated delay schedule which reveal linear waiting more directly than the progressive-ratio schedule (Wynne and Staddon, 1988 see Staddon, 2014, for review).

The finding that the food-deprived condition was associated with higher breakpoints than the free-feeding condition is consistent with many earlier observations of performance on progressive-ratio schedules (Ferguson and Paule, 1997; Hodos, 1961; Hodos and Kalman, 1963; Jenks and Higgs, 2010; Rusted et al., 1998; Skjoldager et al., 1993). This effect has generally been interpreted in terms of a motivation-enhancing effect of deprivation. Whilst the present finding that the value of *a* was higher under the more severe deprivation condition is consistent with this interpretation, it should be noted that, unlike *a*, the breakpoint is sensitive to ‘non-motivational’ manipulations such as the response requirement and the ratio step size (Arnold and Roberts, 1997; Skoljdager et al., 1993; Covarrubias and Aparacio, 2008), and therefore does not constitute a specific index of motivation or incentive value (see Section 1).

### 4.2 Comparison of sucrose and corn oil reinforcers

As reported previously (Olarte-Sánchez et al., 2013), the value of *a* was higher for 25 μl of corn oil than for 50 μl of 0.6 M sucrose in the food-deprived condition. Olarte-Sánchez et al. (2013) noted that although corn oil was evidently more efficacious than 0.6 M sucrose on a volume-for-volume basis, sucrose was the more efficacious reinforcer on a calorie-for-calorie basis. Interestingly, under the free-feeding condition, the values of *a* derived for the two reinforcers did not differ significantly from one another. This suggests that the relationship between deprivation level and reinforcer value may differ between different types of reinforcer.

The values of *δ* did not differ significantly between the sucrose and corn oil reinforcers. However, as previously reported by Olarte-Sánchez et al. (2013), the parameters expressing the minimum post-reinforcement pause (*T*_0_) did differ significantly between the two reinforcers, possibly reflecting the occurrence of more protracted post-prandial orofacial grooming following ingestion of the more viscous reinforcer (see also Bradshaw and Killeen, 2012).

The inclusion of separate parameters to represent response time and post-reinforcement pausing is a feature of the new progressive-ratio model not shared by earlier models derived from MPR, for example the model of performance on fixed-ratio schedules:

(5) $$R_{\text{OVERALL}}=\frac{\zeta }{\delta }-\frac{N}{a}$$

where ζ is a parameter representing the coupling of responses to reinforcers and *a* and *δ* have the same meanings as in Eq. (2) (Killeen, 1994). This equation, which has been applied extensively to performance on progressive-ratio schedules (Bezzina et al., 2008a,b; Covarrubias and Aparicio, 2008; den Boon et al., 2012; Ho et al., 2003; Kheramin et al., 2005; Killeen et al., 2009; Olarte-Sánchez et al., 2012; Rickard et al., 2009; Zhang et al., 2005a,b), defines the maximum response rate as 1/*δ* and makes no allowance for the inclusion of the post-reinforcement pause in the overall response rate. Incorporation of the linear waiting parameters in the new model provides a basis for estimating *δ* without contaminating it with post-reinforcement pausing.

The present results may have some bearing on an ongoing controversy about the sensitivity of the asymptotic response rate in Eq. (4) (*R*_max_) to reinforcer manipulations. According to Herrnstein (1970, 1974), this parameter (*k* according to his nomenclature) represents the totality of behaviour, expressed in units of the reference response. At very high rates of reinforcement, the reference response swamps all other behaviours, causing the rate of measured operant responding to approach its maximum value, *R*_max_. Reinforcer-related variables such as the magnitude or type of reinforcer are assumed to affect the rate of operant responding entirely via changes in the value of *K*_H_, and are not expected to influence *R*_max_ (Herrnstein, 1974). Evidence related to this prediction has been inconsistent, some workers reporting uniform values of *R*_max_ across different sizes and types of reinforcer (Bradshaw et al., 1981; Heyman and Monaghan, 1987, 1994; Petry and Heyman, 1994), and others reporting systematic effects of these variables on the value of *R*_max_ (Belke, 1998; Bradshaw et al., 1978; Dallery et al., 2000; Harper and McLean, 1992; McDowell and Dallery, 1999; Shah et al., 1991). Recent work on the fine structure of responding on variable-interval schedules suggests a way of resolving this difficulty. It has become increasingly evident that overall response rate on these schedules reflects several factors, including the minimum time needed to execute a response, pausing between responses, pausing between bouts of responses, and post-reinforcement pausing (Brackney et al., 2011; Cheung et al., 2012; Killeen et al., 2002; Shull, 2004, 2005; Shull et al., 2004; Smith et al., 2014). All these factors are potentially confounded in the overall response rate (Cheung et al., 2012), and hence in any unitary index of response capacity derived solely from *R*_OVERALL_, such as *R*_max_ in Eq. (4), and *δ* as defined by Eq. (5). Although caution is needed in generalizing findings across different schedules, the successful decomposition of the determinants of maximum response rate in the new progressive-ratio model into response time (*δ*) and post-reinforcement pause time (*T*_0_, *k*) suggests that a similar decomposition of *R*_max_ may be in order. Furthermore, the present finding that *T*_0_ but not *δ* was affected by the quality of the reinforcer raises the possibility that the effects of reinforcer quality on recovered values of *R*_max_ in some previous experiments with variable-interval schedules (Belke, 1998; Bradshaw et al., 1978; Dallery et al., 2000; Shah et al., 1991) may reflect differences in post-reinforcement pausing rather than differences in response time. If this is the case, then fitting Eq. (4) to *R*_RUN_ rather than *R*_OVERALL_ should reduce the effect of reinforcer quality on *R*_max_.

It should be noted that the progressive-ratio model does not take into account the possible contributions of the length of response bouts and the rate of bout initiation to *R*_RUN_. Any such contribution would presumably be absorbed by the recovered value of *δ*, which would therefore need to be further decomposed if the role of response bouts is to be isolated from that of response time (see Brackney et al., 2011). Further work is needed to establish whether the bout-and-pause pattern that characterises variable-interval performance is also a feature of progressive-ratio responding. The present findings offer indirect evidence that this may not be the case, since deprivation level and reinforcer quality did not affect *δ*, whereas such reinforcer-related variables are known to affect bout initiation rate in variable-interval schedules (Brackney et al., 2011; Shull, 2004, 2011; Shull et al., 2004).

There was no significant difference between the breakpoints seen with sucrose and corn oil. This contrasts with the substantial difference between the values of *a* associated with the two reinforcers. However, in a previous study employing identical reinforcers to those used in the present experiment, Olarte-Sánchez et al. (2013) observed higher breakpoints with the corn oil than with the sucrose reinforcer. The reason for this discrepancy is not clear, although, as noted by Rickard et al. (2009), the influence of motivational manipulations on the breakpoint may be less reliable than their effects on *a* when, as in the present experiment, overall response rates are also affected.

### 4.3 Effects of THC

It is well known that THC and other CB1 receptor agonists, including the endocannabinoids anandamide and 2-arachidonoyl glycerol (2-AG), can induce hyperphagia in rats and mice (Brown et al., 1977; Gluck and Ferraro, 1974; Hao et al., 2000; Higgs et al., 2003, 2005; Kirkham and Williams, 2001; Koch, 2001; Williams and Kirkham, 1999). This effect is especially pronounced in the case of sweet and fatty foods (DiPatrizio and Simansky, 2008; Foltin et al., 1988; Higgs et al., 2003, 2005; Jones and Kirkham, 2012; Koch, 2001; Shinohara et al., 2009; Sofia and Knobloch, 1976; Ward and Dykstra, 2005), leading to the suggestion that CB1 receptors may play an important role in determining the incentive values of sapid foodstuffs (Arnone et al., 1997; Higgs et al., 2003, 2005; Simiand et al., 1998; Wakley and Rassmussen, 2009; Williams and Kirkham, 2002a). The ability of CB1 receptor agonists to increase and antagonists to reduce the breakpoint in progressive-ratio schedules has been cited in support of this suggestion (Hernandez and Cheer, 2012; Higgs et al., 2005; Jones and Kirkham, 2012; Maccioni et al., 2008; Rasmussen and Huskinson, 2008; Solinas and Goldberg, 2005; Wakley and Rasmussen, 2009; Ward and Dykstra, 2005).

The progressive-ratio model is well suited to examine the effects of drugs on the incentive value of reinforcers because it allows separate quantification of motivational and motor processes, which are often confounded in univariate indices such as the breakpoint (Bezzina et al., 2015). The present results are consistent with the proposal that CB1 receptors may be involved in determining the incentive values of palatable foods, since acute treatment with THC resulted in a selective increase in the value of *a* for sucrose, none of the other parameters of the progressive-ratio model being significantly affected. As discussed above, a selective increase in the value of *a* is uniquely indicative of an increase in the incentive value of the reinforcer rather than an impairment of motor performance (Bradshaw and Killeen, 2012). In the present experiment, the breakpoint was not significantly affected by THC, suggesting that this index may be less sensitive to THC than the parameter *a*.

A somewhat unexpected finding of this experiment was that THC’s effect on *a* occurred only in the case of performance maintained by the sucrose reinforcer, no effect being apparent in the case of performance maintained by either corn oil (present results) or food pellets (re-analysis of results obtained by Olarte-Sánchez et al., 2012). The food pellets used by Olarte-Sánchez et al. (2012) (TestDiet 5TUM 45 mg pellets) have a low sugar content, the total mono- and disaccharide content amounting to approximately 2.3 mg per pellet (calculated from datasheet: TestDiet, 2011). Taking the relative sweetness of the various sugar constituents into account (Schallenberger, 1993), the sucrose equivalent of a single 45 mg 5TUM pellet is approximately 1.7 mg, compared to 10.27 mg in the case of the sucrose reinforcer used in this experiment (0.6 M, 50 μl). Taken together, therefore, these results suggest that while CB1 receptor stimulation may enhance the reinforcing value of sweet foods, it may have relatively little effect on the value of other foodstuffs.

It is well established that CB1 receptor agonists can enhance the unrestricted intake of both fatty and sweet foodstuffs (DiPatrizio and Simansky, 2008; Koch, 2001); however, less is known about the effects of these drugs on operant behaviour maintained by sweet and fatty reinforcers. Indeed, most previous studies of the effect of these drugs on performance on progressive-ratio schedules used either sucrose or sweetened food pellets as the reinforcer (Hernandez and Cheer, 2012; Higgs et al., 2005; Jones and Kirkham, 2012; Solinas and Goldberg, 2005; Wakley and Rasmussen, 2009). However, the present results are consistent with a report by Ward and Dykstra (2005) that CB1 receptor agonists and antagonists had more pronounced effects on responding maintained by a sweet reinforcer than on responding maintained by corn oil.

The present findings and those of Ward and Dykstra (2005) raise the possibility that the ‘incentive-enhancing’ effect of CB1 receptor agonists may not be entirely attributable to an involvement of these receptors in a general ‘reward system’ (DeLuca et al., 2012; Panagis et al., 2014). There is evidence that CB1 receptors are linked to glutamatergic neurotransmission in the mesocortical/ventral striatal circuit that is believed to regulate the efficacy of divers reinforcers including food, opiates and psychostimulants (Bellocchio et al., 2010). However, CB1 receptors are also present in the taste buds, and stimulation of these receptors selectively enhances the sensation of sweetness (Yoshida et al., 2013; Yoshida and Ninomiya, 2010). The possibility that stimulation of this peripheral receptor population may underlie the selective effect of THC on the incentive value of sucrose may merit further investigation.

## 5 Conclusions

In agreement with previous findings (see above), the results of this experiment indicate that the progressive-ratio model provides a good description of performance on this schedule. The model’s four parameters proffer a means of classifying and quantifying the effects of behavioural interventions and drugs on performance. The sensitivity of the ‘specific activation’ parameter, *a*, to the level of food deprivation and the quality and quantity of reinforcers lends support to the proposal that this parameter is a valid metric of incentive value (Bradshaw and Killeen, 2012; Reilly, 2003). Moreover, the lack of effect of these ‘motivational’ interventions on the ‘response time’ parameter, *δ*, encourages confidence in the utility of this parameter as a measure of ‘motor capacity’ (Killeen, 1994; Bradshaw and Killeen, 2012). An important feature of the model is the decomposition of maximum response rate, allowing post-reinforcement pausing to be treated separately from purely motor constraints on responding. This has enabled the intuitively reasonable attribution of the relatively low maximum overall response rate seen with the more viscous reinforcer (corn oil) to post-prandial behaviours such as orofacial grooming, rather than to motor incapacity (Olarte-Sánchez et al., 2013).

The progressive-ratio model was derived to account for performance on one particular schedule. It is therefore not a competitor of equations with more general applicability, such as Herrnstein’s (1970) response-strength equation. Nevertheless, the benefits derived from decomposing the maximum response rate in the progressive-ratio model suggests that a similar manoeuvre may be in order in the case of Herrnstein’s (1970) equation (Bradshaw, 1994). It is suggested that this may help to resolve the ongoing controversy about the sensitivity (or otherwise) of *R*_max_ to motivational manipulations (Heyman and Monaghan, 1987; McDowell, 2013).

Finally, the effect of THC seen in this experiment suggests that this drug may preferentially enhance the incentive value of sweet tasting reinforcers (Ward and Dykstra, 2005). The possibility that this may reflect an effect of THC on peripheral taste receptors needs further investigation.

## Figures and Tables

**Table 1:** Sucrose reinforcer: effects of THC on the parameters of the progressive-ratio model, and the breakpoint, in rats maintained under the food-deprived condition (group mean values ± SEM).

Parameter	Vehicle	THC 0.3 mg kg^−1^	THC 1 mg kg^−1^	THC 3 mg kg^−1^
*T*_0_, s	3.33 ± 0.29	3.72 ± 0.65	2.62 ± 0.68	3.19 ± 0.34
*k*	0.51 ± 0.03	0.53 ± 0.04	0.54 ± 0.04	0.48 ± 0.05
*a*, s	23.4 ± 5.4	24.4 ± 5.4	31.1 ± 5.6^a^	30.2 ± 7.4^a^
*δ*, s	0.21 ± 0.02	0.2 ± 0.03	0.25 ± 0.03	0.24 ± 0.02
*R*^2^	0.94 ± 0.01	0.9 ± 0.03	0.91 ± 0.02	0.92 ± 0.02
Breakpoint	163.3 ± 38.8	170.5 ± 38.8	176.3 ± 43.1	157.1 ± 31.6

a Significantly different from vehicle control condition, *P* < 0.05.

**Table 2:** Corn oil reinforcer: effects of THC on the parameters of the progressive-ratio model, and the breakpoint, in rats maintained under the food-deprived condition (group mean values ± SEM).

Parameter	Vehicle	THC 0.3 mg kg^−1^	THC 1 mg kg^−1^	THC 3 mg kg^−1^
*T*_0_, s	5.63 ± 1.33	5.62 ± 1.29	6.14 ± 1.56	6.35 ± 1.56
*k*	0.63 ± 0.09	0.62 ± 0.09	0.58 ± 0.1	0.56 ± 0.1
*a*, s	70.2 ± 17.7	73 ± 19.7	64.3 ± 18	67.7 ± 18.6
*δ*, s	0.44 ± 0.15	0.47 ± 0.12	0.37 ± 0.12	0.45 ± 0.15
*R*^2^	0.88 ± 0.03	0.86 ± 0.04	0.84 ± 0.04	0.81 ± 0.05
Breakpoint	173.7 ± 46.5	166.4 ± 46.7	177.3 ± 48	152.4 ± 38.7

**Table 3:** Food-pellet reinforcer: Effects of THC on the parameters of the progressive-ratio model, and the breakpoint, in rats maintained under the food-deprived condition (group mean values ± SEM).

Parameter	Vehicle	THC 0.3 mg kg^−1^	THC 1 mg kg^−1^	THC 3 mg kg^−1^
*T*_0_, s	4.81 ± 0.94	5.92 ± 1.2	6.14 ± 1.17	4.5 ± 0.56
*k*	0.5 ± 0.06	0.51 ± 0.06	0.44 ± 0.05	0.51 ± 0.07
*a*, s	28.1 ± 4.8	26.8 ± 4.9	26.2 ± 5.4	26.5 ± 5.1
*δ*, s	0.23 ± 0.03	0.2 ± 0.02	0.2 ± 0.02	0.23 ± 0.02
*R*^2^	0.96 ± 0.01	0.93 ± 0.02	0.94 ± 0.01	0.93 ± 0.02
Breakpoint	127.4 ± 18.2	126.1 ± 19.6	134 ± 19.9	127.4 ± 17.5

## Footnotes

1 This nomenclature differs from that used by Herrnstein (1970). *R*_max_ and *K*_H_ are algebraically identical to Herrnstein’s (1970)*k* and *r*_o_; however, unlike Herrnstein’s parameters, *R*_max_ and *K*_H_ are theoretically neutral. *R*_max_ expresses the maximum reponse rate and *K*_H_ the reinforcement rate corresponding to *R*_max_/2 (Bradshaw et al., 1976; Shah et al., 1991; for discussion, see McDowell, 2013). In most experimental applications of Eq. (4), *R* has been taken to refer to *R*_OVERALL_ (see text for further discussion). It should be noted that the term k in Eq. (1) is not related to the parameter of the same name in Herrnstein’s (1970) model.
